# Improving the outcomes of foot and ankle surgery through the audit cycle: a case study

**DOI:** 10.1186/1757-1146-4-S1-O21

**Published:** 2011-05-20

**Authors:** Rob Hermann, Paul Bennett, Danielle Gallegos

**Affiliations:** 1 School of Public Health, Queensland University of Technology, Brisbane, Queensland, 4059, Australia

## 

The direct costs of managing adverse outcomes from Australian health care are estimated to be $2 billion. The audit cycle is considered an important tool to assist in the preventive management of adverse outcomes.Australian guidelines for audit cycle design allow for comparison of data sets derived from similar surgical specialities. However a lack of data set standardisation inhibits meaningful comparisons of foot and ankle surgical audits. This research will assist development of a best practice model for auditing foot and ankle surgery. Data derived from this model will improve the safety and quality of foot and ankle surgery. The preliminary phase of this process is to identify and understand the attitudes and behaviours of *how* and *why* surgeons participate in the audit cycle. A descriptive embedded multiple case study research design is planned to provide an intense focus on a single phenomenon (the audit cycle) within its real life context (clinical governance). The measures to be included in the case study have been identified by the Balanced Patient Safety Measurement Framework. These include: audit and peer review activity, provider attitudes to patient safety, safety learning, action and performance. A purposive sample of 6 to 8 surgeons (units of analysis) from 3 to 4 specialities (cases) will undergo semi-structured interview. This will investigate: current audit tools and processes; attitudes; and behaviours of surgeons to the audit cycle. Similarities in and differences between the units of analysis will indicate which identified measures function as barriers or enablers of the audit cycle. Reliability and validity (external and construct) will be assessed using established methods for case studies. The descriptive embedded multiple case study will reveal *how* and *why* foot and ankle surgeons participate in the audit cycle. This will inform further research to improve the outcomes of foot and ankle surgery through development of an audit tool.

